# Management of *Helicobacter pylori* infections

**DOI:** 10.1186/s12876-016-0496-2

**Published:** 2016-08-12

**Authors:** Amin Talebi Bezmin Abadi, Johannes G. Kusters

**Affiliations:** 1 Medical Bacteriology, Faculty of Medical Sciences, Tarbiat Modares University, Tehran, Iran; 2 Department of Medical Microbiology, University Medical Center Utrecht, Heidelberglaan 100, Utrecht, 3584 CX The Netherlands

**Keywords:** *H. pylori*, Eradication therapy, Gastric cancer, Antimicrobial resistance

## Abstract

**Background:**

Infection with *Helicobacter pylori* is associated with severe digestive diseases including chronic gastritis, peptic ulcer disease, and gastric cancer. Successful eradication of this common gastric pathogen in individual patients is known to prevent the occurrence of peptic ulcer disease and gastric cancer.

**Discussion:**

With half of the world’s population being infected with *H, pylori* and only few antibiotics result in an effective eradication, a successful antibiotic driven worldwide eradication program seems unlikely. In addition, *H. pylori* eradication is not always beneficial as it has been described that eradication can be associated with an increased frequency of other disorders such as pediatric asthma, inflammatory bowel diseases and Barrett’s Esophagus. We have to accept that eradication of this infection is a two-edged sword that is both useful and harmful and we should therefore focus our *H. pylori* eradication policy toward selectively identify and destroy only the virulent strains.

**Conclusion:**

In order to still be able to effectively treat *H. pylori* infections in the future we need an alternative diagnostic/treatment algorithm. This would involve a shift towards more precise and enhanced disease predicting diagnosis that tries to identify patients with chance of developing severe diseases such as gastric cancer, rather than the current regime that is geared towards find and destroy all *H. pylori*.

## Background

**Fig. 1:**
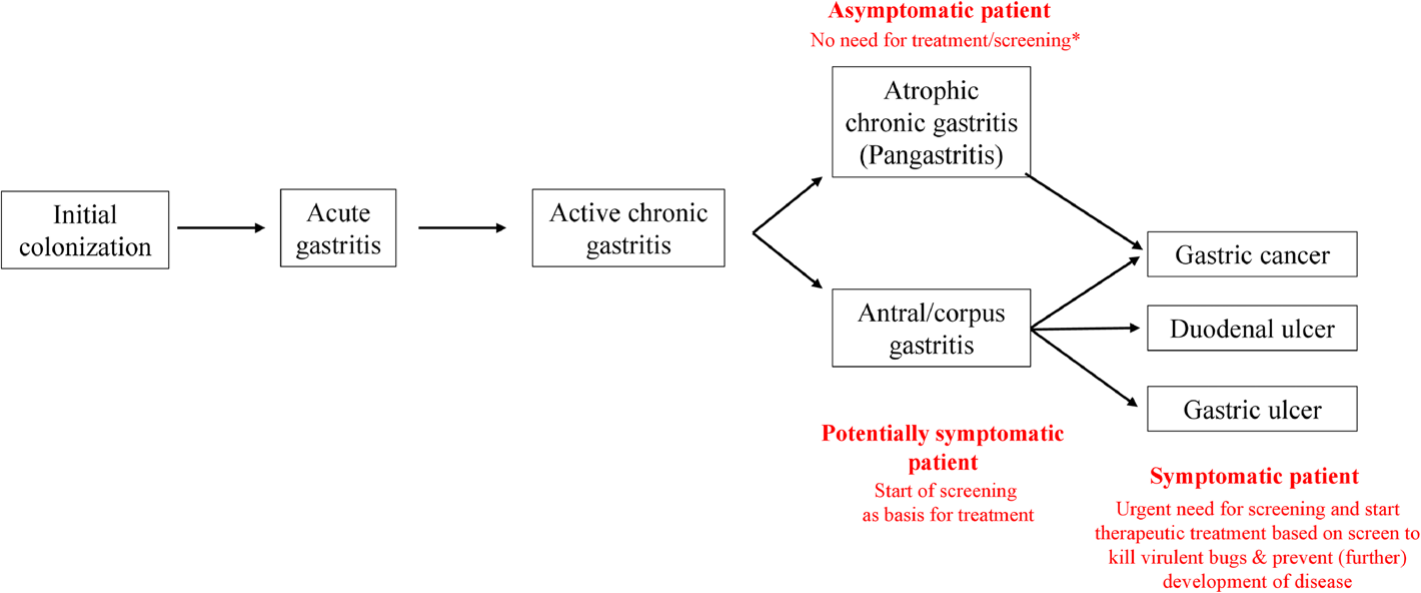
Stopping the Helicobacter pylori induced disease cascade. Depending on the host and bacterial characteristics the initial colonization will result in gastritis and this will either result in clinical symptoms (symptomatic patient) where screening for the presence of *Helicobacter* and its resistance needs to be performed in order to allow for rational treatment. If the infection does not result in clinical symptoms (asymptomatic patient) it may still be advisable to use a single non-invasive fecal antigen screen at age 40 or so (when usually symptoms have not yet developed) to test for the presence of *Helicobacter*. When this test is positive fecal DNA test detecting *Helicobacter* specific virulence factors to predict infection with a virulent strain, and/or a noninvasive serologic follow-up screen to establish the degree of atrophy is advisable. If either of these tests predict there is a high risk for the development of gastric cancer eradication is advisable. Elimination of strains that contain virulence markers associated with an increased risk for disease development (regardless of what pathogenicity - if any it is causing in its current host) will l reduce the spread of virulent strains and thereby force the population of circulating *Helicobacte*r isolates into less pathogenic *Helicobacters, irrespective of the contribution of host genetics and environmental factors*

With >50 % of the world’s population colonized by *Helicobacter pylori* it is one of the most prevalent human pathogens [1]. Infection with *H. pylori* is associated with chronic gastritis, duodenal ulcer, gastric cancer and even gastric adenocarcinoma [2]. Infection is usually acquired in childhood via person-to-person fecal-oral, oral-oral, or gastro-oral transmission being the most likely route of infection [3]. When ingested the bacterium colonizes the mucous layer covering the epithelial cells of the stomach and this induces an immune response that in spite of its vigor is unable to eliminate the bacterium [4]. The chronic infection that results from this vigorous but typically inadequate immune response induces gastric damage that is associated with digestive disorders ranging from chronic gastritis to peptic ulcer disease, and gastric cancer, but in most cases *H. pylori* infected patients remain asymptomatic for a long time [5]. In contrast to many other infectious agents the final outcome of a *H. pylori* infection is different for each infection and is dependent on both host and bacterial factors [1]. The large genetic variation observed among clinical strains of *H. pylori* is thought to be an important determinant in the specific local establishment of this colonization and determines in large part the development of the potential disorders that result from the infection [6]. The remarkable genetic and phenotypic diversity observed in *H. pylori* is thought to be driven by the need to adapt to the harsh and patient specific conditions of the gastric environment that often result in the genesis of various subpopulations (quasispecies) within single patients [7]. This results in each infected person potentially carrying genetically unique microorganisms existing only in that particular patient [7, 8]. Depending on the presence of specific *H. pylori* virulence factors such as *dupA*, *cagA*, *vacA* and *homB* the bacterium usually colonizes the antral mucosa of human stomach [8]. Once this primary colonization is established it induces an inflammation that remains largely asymptomatic (Fig. 1) [9, 10]. In fact, an acute infection and subsequent gastritis precede the more severe digestive disorders such as gastric bleeding, peptic ulcers, and gastric cancer known to be associated with *H. pylori* infection (Fig. 1) [10]. While a long list of antibiotics has been tested for their effectiveness on *H. pylori* only a handful of them can be used for effective *H. pylori* eradication. Treatment options are further reduced as effective therapeutic regimen requires a combination of two or three antibiotics and a gastric acid suppressive drug [11]. Finally, in *H. pylori* antibiotic resistance to these drugs rapidly emerges as the resistance against all these drugs does not require the introduction of specific genes, but in all cases is based on a single point mutation in an existing housekeeping gene thus and obviously this further reduces a broad clinical efficacy significantly [4, 12, 13]. In parallel with emergence of multidrug resistant bacteria in other bacterial species, we need to accept that there is a rapid and ongoing reduction of the effective lifespan of antibiotics for clinical applications [14]. This has resulted in a situation where the big companies stopped or reduced their efforts to develop new antibiotics. While vaccination may be a practical approach for many pathogens, in the case of *H. pylori* vaccines do not seem to represent a viable option as sterilizing immunity is not considered an endpoint for now [15, 16]. As currently vaccines and pre/probiotics do not offer realistic *H. pylori* eradication strategies antibiotic treatment is currently the only available option to effectively reduce the gastrointestinal disorders resulting from *H. pylori* infection [17–19]. For now the further increase of antimicrobial resistance in *H. pylori* is being endangered by clinicians that frequently prescribe clarithromycin for patients suffering from upper respiratory diseases. Especially in developing countries many of these respiratory disease patients will also be colonized with *H. pylori,* but as here *H. pylori* infection is still in its early stage it will probably not have resulted in any significant disease symptoms and thus go unnoticed. Treating these patients with antibiotics with what should be considered for *H. pylori* as low dose, relative short period, and in the absence of an acid suppressive agent will not eradicate the *H. pylori* colonization but instead is very likely to promote the generation of resistant *H. pylori.* Hence we would need to create the awareness amongst clinicians that they might need to save the few remaining effective *H. pylori* drugs such as clarithromycin and amoxicillin and not use them freely for the treatment of common respiratory and urinary tract infections because of the consequences this may have on future *H. pylori* treatment of their patients. Or in minimum test for the absence of *H. pylori* in these patients and refrain from the use of effective *H. pylori* drugs when a patient is positive.

## Discussion

While many diagnostic tests for detecting *H. pylori* are expensive and/or time consuming, our above suggestion to apply molecular detection will probably not get much support. Especially since currently when treating *H. pylori* infections more and more eradication therapies are being prescribed without testing for antimicrobial sensitivity in order to save costs [17]. Effectively this means that often a combination therapy is prescribed without propper sensitivity testing. Due to increased resistance rates this may in fact effectively boil down to prescribing monotherapy. We know that in those cases treatment will not only be less effective, but in fact favors the generation of resistance to the one drug that was effective. While currently invasive procedures are still required in order to obtain material for culture and antimicrobial resistance determination, there is a now molecular method being developed that may allow for fecal matter based PCR testing for resistance against many of the commonly used *H. pylori* drugs [20, 21]. It will be relatively easy to also include a selection of *H. pylori* virulence markers in these tests, facilitating screening algorithm that would allow discriminating the good *H. pylori* from the bad. This would give rise to the screening and eradication scheme outlined in Fig. 1, where in principle only symptomatic patients would be treated after testing for virulence and resistance. Only anti-*H. pylori* therapies that can achieve eradication rate ≥90 % are considered effective, but increasing resistance hampers the efficacy of prescribed regimens [12, 21, 22]. Up to now we have taught clinicians to eradicate all cases of *H. pylori* infections as they would with any other pathogenic bacterial infection [23, 24]. However in the light of our current knowledge where only a minority of *H. pylori* infections will cause clinical symptoms screening for *H. pylori* and subsequent eradication may no longer be the best approach. For one, recent studies showed that asthma and other diseases can appear in the absence of *H. pylori*, an interesting phenomenon which complicates links between *H. pylori* infection and human disease [25, 26]. However we should consider occurrence of gastric cancer while it can be reduced in *H. pylori* infected patients through the elimination of *H. pylori* (either by antibiotic therapy or vaccine) [27, 28]. Current findings are indicate an association between *H. pylori* free individuals and certain immune malfunctional diseases including allergy and asthma [26, 29], inflammatory bowel diseases (IBS) [30, 31] and Barrett’s Esophagus (BE) [32] in Helicobacter free patients. One has to be aware however that this protective link between *H. pylori* infections and immune related disorders is not always observed and thus disputed by some authors [33]. While infection with *H. pylori* will result in gastritis subsequently we should be aware that only very few infected patients, even when left untreated will actually develop gastric cancer. Basically, an Operative Link for Gastritis Assessment (OLGA)-staging system has been designed to simplify evaluation of gastric atrophy [34]. This staging system was an updated version of Sydney system, thus, the staging system can then rank the histologic phenotypes of gastritis with a progressively increasing gastric cancer risk [35]. Moreover, new challenge can be to develop different algorithms that would predict people with high risk of developing gastric cancer using the pathotype of the infecting strain of *H. pylori*. In this proposed algorithm, only virulent *H. pylori* strains should be targeted/eliminated, thus allowing non-virulent strains to remain in place and so that they can exert their beneficial effects. With Next Generation Sequencing (NGS) techniques slowly becoming a routine technique in human diagnostic laboratories and its prices and turn-around times rapidly falling finding and destroying only virulent *H. pylori* strains may soon become a viable option. The classical perception that all infections should be treated as potentially disease causing has promoted that clinicians were eager to eliminate *H. pylori* infections and that is why they used (or in our view perhaps massively overused) antibiotics to eradicate this infection.

Perhaps for the control of future symptoms in the currently asymptomatic *H. pylori* patients we should introduce a population based screen for the presence of *H. pylori* at age 50 using a non-invasive test like the fecal antigen test or urease breath tests to establish the presence of a *H. pylori* infection (Fig. 1) [36]. For those positive follow-up screening with either non-invasive molecular test that establishes the virulence of the infecting strain (see above), and/or a serologic tests that would predict the status of the atrophy [37]. If these tests would predict an increased risk of future disease development, treatment would then be indicated, and one would proceed as outlined above for the symptomatic patient. The problem of *H. pylori* antibiotic resistance is real and as a result, we are now confronted with a new generation of multi drug resistant *H. pylori* strains, for which suitable treatment options no longer exist.

## Conclusion

Thus we may need to revise the available guidelines to include the option where the best possible treatment of an infection may no longer be geared towards blindly eradicating all infected patients (search and destroy) but be directed towards a selective eradication strategy (find and destroy only virulent strains). However this requires the implementation of effective strategies to identify virulent strains as well as awareness amongst physicians that treatment of all individual patients may severely limit the treatment options for our future patients.

## Abbreviations

BE, Barrett’s esophagus; IBS, inflammatory bowel diseases; NGS, next generation sequencing; OLGA, operative link for gastritis assessment
